# USV-Observed Turbulent Heat Flux Induced by Late Spring Cold Dry Air Incursion over Sub-Mesoscale Warm Regions off Sanriku, Japan

**DOI:** 10.3390/s22249695

**Published:** 2022-12-10

**Authors:** Akira Nagano, Takuya Hasegawa, Keisuke Ariyoshi, Takeshi Iinuma, Tatsuya Fukuda, Nobuhiro Fujii, Fumiaki Tomita, Ryota Hino

**Affiliations:** 1 Research Institute for Global Change, Japan Agency for Marine-Earth Science and Technology (JAMSTEC), Yokosuka 237-0061, Japan; 2 Faculty of Environmental Earth Science, Hokkaido University, Sapporo 060-0808, Japan; 3 Graduate School of Science, Tohoku University, Sendai 980-8577, Japan; 4 Research Institute for Marine Geodynamics, Japan Agency for Marine-Earth Science and Technology (JAMSTEC), Yokohama 236-0001, Japan; 5 Institute for Marine-Earth Exploration and Engineering, Japan Agency for Marine-Earth Science and Technology (JAMSTEC), Yokosuka 237-0061, Japan; 6 Marine Works Japan Ltd., Yokosuka 237-0063, Japan; 7 International Research Institute of Disaster Science, Tohoku University, Sendai 980-8572, Japan; 8 Research Center for Prediction of Earthquake and Volcanic Eruptions, Graduate School of Science, Tohoku University, Sendai 980-8578, Japan

**Keywords:** warm-core ring, air–sea interaction, turbulent heat flux, sub-mesoscale variation, Wave Glider

## Abstract

We performed oceanic and atmospheric observations in the region off the Sanriku coast, Japan, from May 11 to 5 July 2022, using a wave-propelled unmanned surface vehicle, a Wave Glider (WG). Despite the severe weather conditions of atmospheric low-pressure system crossings, we successfully measured wind, air temperature, humidity, and sea surface temperature over the course of 55 days to calculate the turbulent heat flux. The WG observed that the atmosphere became more humid due to the southerly wind along the northwestern rim of the North Pacific subtropical high. The warm Kuroshio water expanded to the southeast of Hokkaido as a result of the northward shedding of an anticyclonic mesoscale (~100 km) eddy, called a warm-core ring, from the Kuroshio Extension. The WG traversed smaller (sub-mesoscale) water regions that were warmer and saltier than the surrounding Kuroshio water. The observations indicate that cold, dry air masses advected by northerly winds following the passage of atmospheric low-pressure systems generate a substantial upward turbulent heat flux over sub-mesoscale warm water regions, contrasting to no heat flux in the surrounding Kuroshio water region.

## 1. Introduction

**Figure 1:**
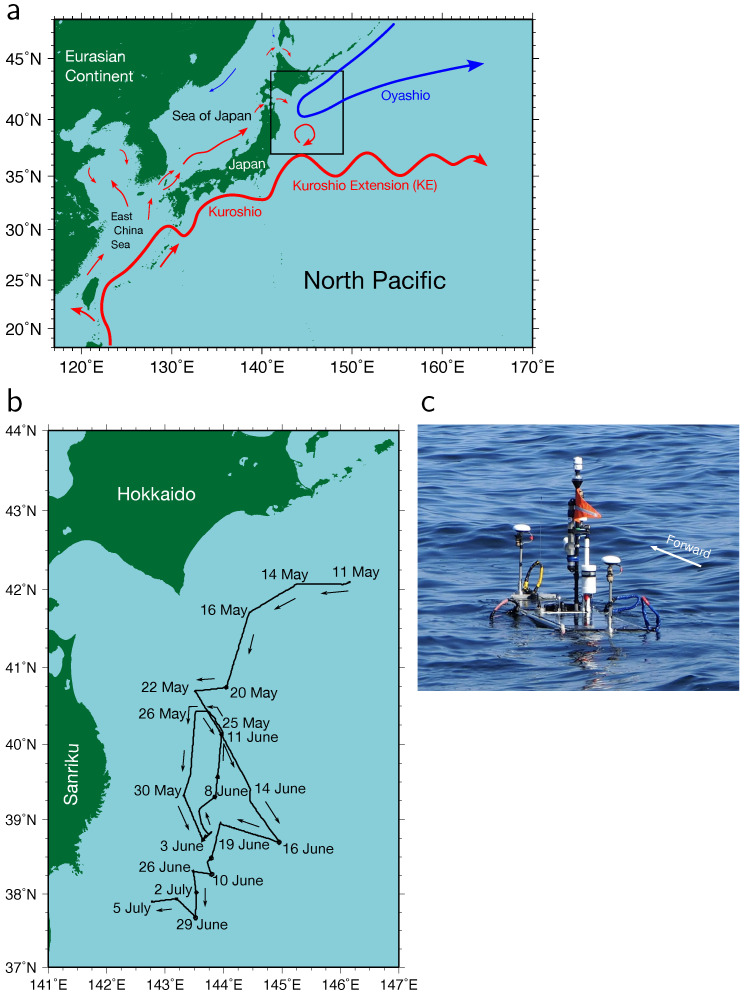
(**a**) Schematic diagram of the subtropical (red arrow lines) and subpolar (blue arrow lines) surface currents in the western North Pacific. The experiment region enclosed by the square is depicted by the enlarged map in panel (**b**). (**b**) Wave Glider (WG) track (black line) and passage dates. The directions of the WG are illustrated by arrows. (**c**) The observing WG immediately after the deployment on 11 May 2022.

A vast amount of heat is transported northward from the tropics to the subtropics via the Kuroshio, the western boundary current of the North Pacific subtropical gyre e.g., [1,2,3], which originates in the region east of the Philippines, e.g., [4] (Figure 1a). Thus, the sea surface temperature (SST) in the Kuroshio and Kuroshio Extension (KE) regions is extremely high compared to the surrounding regions, and heat is actively released into the atmosphere throughout the boreal winter as turbulent latent and sensible heat fluxes, e.g., [5,6]. The huge amount of heat released in the winter contributes to the global heat balance, e.g., [1]. Therefore, turbulent heat fluxes are key parameters for considering the role of the ocean in the climate system. In other seasons, a smaller but significant amount of heat is considered to be released to the atmosphere if the atmosphere and ocean are in favorable conditions. The released heat may have effects on Japan’s regional climate and the weather. Knowledge on the turbulent heat flux variation leads to the improvement of climate and weather forecasts.

The Oyashio, the western boundary current of the North Pacific subpolar gyre, flows southward in the region off the Hokkaido coast, Japan, (Figure 1a) and frequently proceeds to the east of the Sanriku coast. The Oyashio current transports fresh cold water to the eastern regions of Japan. The area between the Kuroshio and Oyashio regions is referred to as the perturbed area due to the complicated distribution of waters from the Kuroshio and Oyashio regions by mesoscale (30–100 km) eddies and fronts [7]. Strong mesoscale anticyclonic eddies, known as warm-core rings (WCRs), are occasionally detached from the KE meander’s crest and intrude into the perturbed region (Figure 1a). The WCRs retain the Kuroshio water, characterized by high temperature and salinity, within their interiors, thereby exhibiting temperature and salinity contrasts in the region off the Sanriku coast. Furthermore, there may be smaller scale (sub-mesoscale) variations derived from the WCRs. The SST contrasts caused by mesoscale and sub-mesoscale variations have effects on the surrounding environment by generating circulations in the atmospheric boundary layer through the heat released into the atmosphere.

Lately, ocean mesoscale and sub-mesoscale variations have been mainly observed by satellites. By the state-of-the-art techniques, satellite observations provide high-resolution turbulent heat flux data with a horizontal resolution of $0.25^{{}^{\circ}}$, which can resolve heat flux variations due to mesoscale eddies in the Kuroshio and KE regions [8]. However, certain variables, such as SST observed by satellites, are frequently obscured by clouds and other factors in rainy seasons even when the measurement resolutions are sufficiently high in space and time. In particular, sub-mesoscale variations in all the parameters required to estimate turbulent heat fluxes have not been observed in the perturbed region. Therefore, it is unclear how the oceanic sub-mesoscale variations affect the atmospheric boundary layer. In situ observations are important to accurately understand mesoscale and sub-mesoscale eddies, fronts, and their effects on the atmospheric boundary layer. However, massive research vessels could disrupt the boundary layers of the atmosphere and ocean. Smaller platforms are preferable to obtain accurate measurements of the variables required to estimate the turbulent latent heat flux (LHF) and sensible heat flux (SHF).

Unmanned surface vehicles (USVs) have been developed, e.g., [9], and are useful for observing atmospheric and oceanic parameters with high spatiotemporal resolutions and accuracy sufficient to support scientific research. For example, Nagano and Ando [6] observed the atmosphere and ocean across a mesoscale warm spot in the Kuroshio south of Japan using a Saildrone, a wind-powered USV (https://www.saildrone.com, accessed on 9 December 2022). They discovered smaller scale spatial structures of SST and the corresponding wind variation over the mesoscale high SST region. By using ocean-wave-propelled USVs, called Wave Gliders (WGs) (https://www.liquid-robotics.com, accessed on 9 December 2022), and a research vessel, Nagano et al. [10] observed variations in atmospheric and oceanic variables on time scales of days in the eastern region of the western tropical Pacific warm pool. Observations by USVs can reveal sub-mesoscale variations in SST and other variables at the sea surface layer.

Wind speed, relative humidity, air temperature, and SST are required to estimate turbulent heat flux based on a bulk flux algorithm, e.g., [11,12]. Of these atmospheric and oceanic parameters, measurements of humidity over the sea are especially difficult because sensors are not designed for use at sea and are easily damaged by seawater immersion. Nevertheless, humidity is a particularly important parameter in the Kuroshio and KE regions throughout the year, associated with the Baiu (Japan’s rainy season) onset and withdrawal in late spring to early summer, typhoons and heavy rainfall in summer to autumn, and cold dry winds from the Eurasian Continent in winter to early spring.

To observe the variation in the turbulent heat flux on spatial scales down to the sub-mesoscale in the perturbed region, we performed a field experiment by deploying a WG in the region off the Hokkaido coast on 11 May 2022, observing for 55 days, and recovering it on 5 July 2022. To obtain reliable humidity data throughout the observation period, we protected humidity and air temperature sensors by covering them with pieces of waterproof breathable fabric, as described below. We measured all the parameters required to estimate turbulent heat flux from the bulk flux algorithm during severe weather conditions caused by the passage of several atmospheric low-pressure systems over the WG, and as a result, we obtained turbulent heat fluxes east of the Sanriku coast. In particular, we successfully observed sub-mesoscale warm waters in the perturbed area for the first time. Additionally, we observed an increase in specific humidity during the observation period both before and during Japan’s rainy season, i.e., the Baiu season, accompanied by moistened southerly winds and substantial heat release from the ocean to the atmosphere caused by occasional occurrences of dry cold northerly winds over the sub-mesoscale warm regions.

The remaining sections of this study are structured as follows. The observation and configuration of the WG are described in Section 2. In Section 3, we calculate turbulent heat flux based on the parameters observed by the WG and, using satellite and reanalysis data, identify the parameters and conditions where the heat flux is enhanced. The summary and conclusions are provided in Section 4.

## 2. Observation and Data

In addition to the primary object of the WG observation, which was the observation of crustal deformation using the global navigation satellite system-acoustic (GNSS-A) technique as reported by Iinuma et al. [13], we installed meteorological and oceanographical sensors on the WG and measured atmospheric and oceanic variables. The USV used in this experiment is a Wave Glider (version SV3) (Liquid Robotics, Sunnyvale, CA, USA; https://www.liquid-robotics.com, accessed on 9 December 2022), which is a form of autonomous vehicle powered by waves [14,15]. The underwater glider, which is connected to the float by an 8-m umbilical cable, converts wave energy into forward motion. The float has a length and width of 3.05 and 0.81 m, respectively. Details of the WG used in this experiment are provided by Iinuma et al. [13]. The WG was deployed in the region southeast of Hokkaido on 11 May 2022 at 07:40 (Japanese standard time [JST]) by the research vessel (R/V) *Kaiyo Maru No. 3* (Kaiyo Engineering Co., Ltd., Tokyo, Japan) and recovered on 5 July 2022 at 05:40 (JST) by the R/V *Kaiyo Maru No. 2* (Kaiyo Engineering Co., Ltd., Tokyo, Japan). During the observation, the GNSS determined the location of the WG with a 2-m uncertainty radius. The WG’s path is depicted in Figure 1b. The WG observed meteorological and oceanographic data along the path, occasionally staying at GNSS-A stations to conduct the observations of crustal deformation.

**Figure 2:**
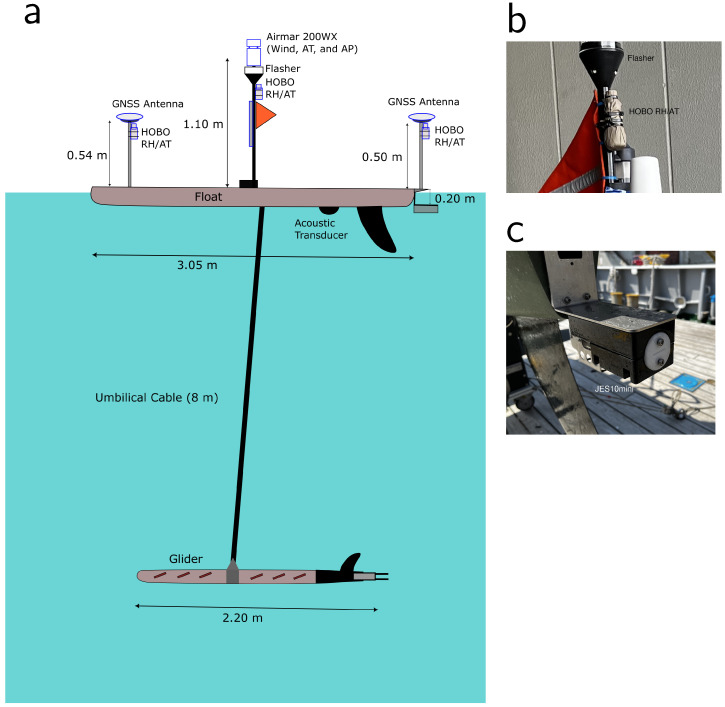
(**a**) Diagram of a Wave Glider SV3. Images of (**b**) the HOBO data logger on the middle mast of the WG and (**c**) the CTD JES10mini under the tail of the WG float. The HOBO sensor in panel (**b**) is covered by beige Gore-Tex fabric.

A conductivity-temperature-depth (CTD) JES10mini (Offshore Technologies, Yokohama, Japan) was installed at a depth of 0.2 m under the WG’s float (Figure 2) to collect temperature and salinity data at 1-min intervals. The temperature and salinity accuracies of the CTD are ±0.005${}^{{}^{\circ}}$C and ±0.005 $S\;m^{-1}$, respectively. The accuracy of salinity is better than the ±0.04 (practical salinity scale). The salinity readings declined commencing around 15 June 2022, to a minimum of 31.5, despite being in the salt-rich Kuroshio region, and then increased to over ~33 on 26 June 2022. This abnormally low salinity may have been caused by a goose barnacle stuck on the inside of the glass tube containing the electrodes, which inhibited the intake of seawater. Accordingly, the salinity data after 15 June 2022 were omitted from the subsequent analysis.

A 200WX Weather Station (AIRMAR Technology Corporation, Milford, NH, USA) was equipped to the middle mast of the WG at a height of 1.1 m (Figure 2), and air temperature, barometric pressure, wind direction, and speed were observed at 10-min intervals. Additionally, we installed three HOBO U23-001A Pro v2 data loggers (Onset Computer Corporation, Bourme, MA, USA) to the head (0.5 m height), middle (0.9 m height), and tail (0.5 m height) masts, and we observed air temperature and relative humidity at every 5-min interval. The accuracies of temperature and relative humidity were ±0.2${}^{{}^{\circ}}$C and ±2.5%, respectively. The information of these meteorological and oceanographic instruments is listed in Table 1.

We doubly covered the HOBO sensors with commercially available Gore-Tex (W.L. Gore & Associates, Inc., Flagstaff, AZ, USA) fabric to prevent seawater from damaging the humidity sensors. The Gore-Tex fabric used in this experiment features a three-layered structure with a waterproof, breathable membrane of expanded polytetrafluoroethylene sandwiched between chemical synthetic fabrics. An examination by use of a diode laser spectroscope shows that Gore-Tex fabric is quite breathable for water vapor [16]. Although the WG was once overturned after deployment, relative humidity sensors continued to function properly throughout the observation.

**Figure 3:**
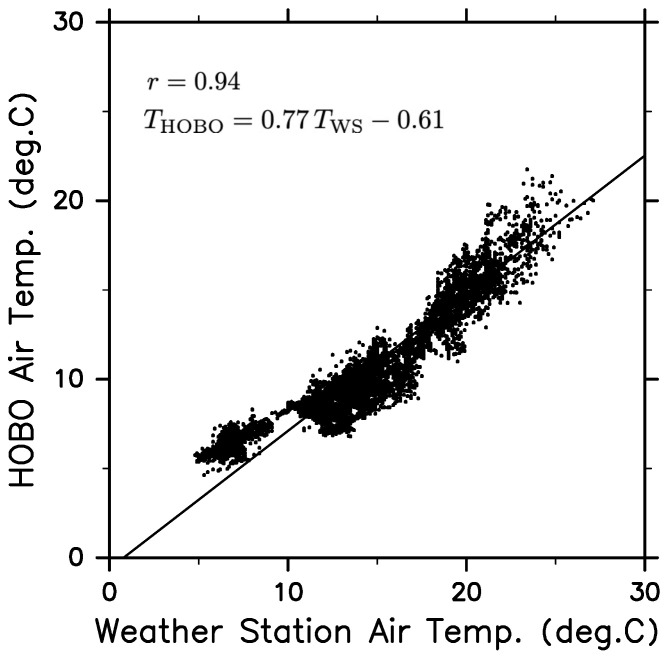
Scatter plot of observed temperature values by Weather Station sensor ($T_{\mathrm{WS}}$) versus those by HOBO sensor ($T_{\mathrm{HOBO}}$). Their correlation coefficient is r=0.94. The linear regression equation ($T_{\mathrm{HOBO}}=0.77\;T_{\mathrm{WS}}-0.61$) is indicated by the slanted line.

Temperatures inside and outside the fabric are assumed to change by $\Delta T_{i}$ and $\Delta T_{o}$, respectively, while air moves through the Gore-Tex fabric at an exchange rate of *E* for a duration of Δt. Considering the heat balance inside the fabric of volume *V*, we obtain the ratio of $\Delta T_{i}$ to $\Delta T_{o}$, i.e., $\Delta T_{i}/\Delta T_{o}=1/\left(1+V/\left(E\Delta t\right)\right)$. In the absence of fabric, we expect *E* to be almost infinite, implying that the ratio would be infinitely close to the unity. We compared the temperatures collected by the Weather Station and the HOBO sensors. We observed a correlation coefficient of r=0.94 (Figure 3), which is significantly higher than the 99% confidence level, and the root-mean-square error was 1.55${}^{{}^{\circ}}$C. The regression slope (0.77), which is equivalent to $\Delta T_{i}/\Delta T_{o}$, is less than one. Thus, the exchange of air may be reduced by a finite *E* due to the existence of the Gore-Tex fabric. The same argument may apply to the moisture concentration, but we did not measure the relative humidity outside the fabric. If our guess is correct, the humidity variation observed within the fabric may be approximately 33% lower than expected. Nevertheless, the HOBO humidity measurements are useful for monitoring variations in humidity.

We computed LHF and SHF by applying the COARE3.0b bulk flux algorithm developed by Fairall et al. [12] to atmospheric and oceanic variables collected every 10 min by the WG. We used temperature data from the JES10mini CTD sensor as SST data to calculate heat fluxes rather than performing the skin temperature correction, as the shortwave and longwave radiation fluxes were not measured.

Daily $1/4^{{}^{\circ}}$× 1/4${}^{{}^{\circ}}$ gridded SST data from May to July 2022 (NOAA OI SST V2 High Resolution Dataset) provided by Reynolds et al. [17] were used to monitor horizontal SST distribution in the study region spanning 37–$44^{{}^{\circ}}$ N, 141–$147^{{}^{\circ}}$ E. We employed the sea-level pressure field in the region of 30–$45^{{}^{\circ}}$ N, 130–$160^{{}^{\circ}}$ E obtained from $1.25^{{}^{\circ}}\times 1.25^{{}^{\circ}}$ gridded data from the Japanese 55-year Reanalysis (JRA-55) conducted by the Japan Meteorological Agency [18].

## 3. Results and Discussion

**Figure 4:**
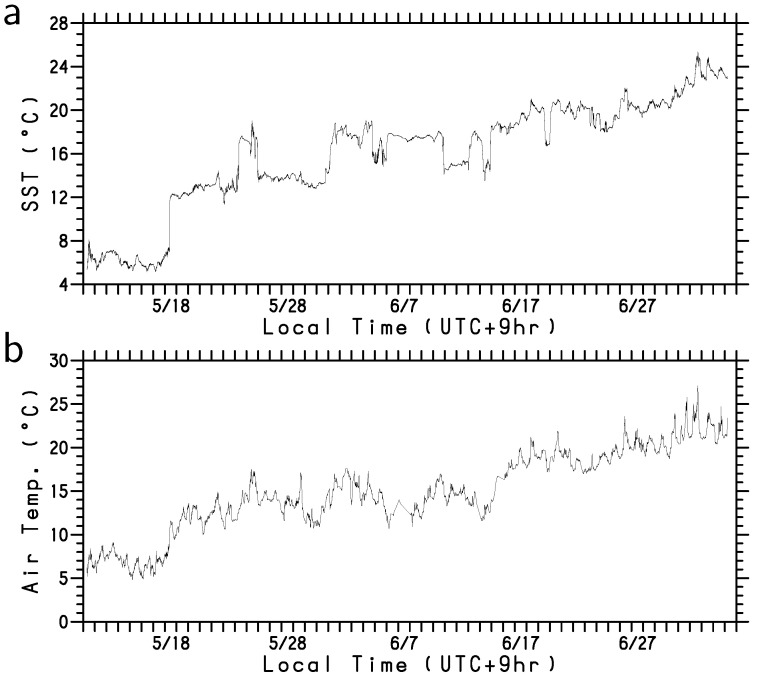

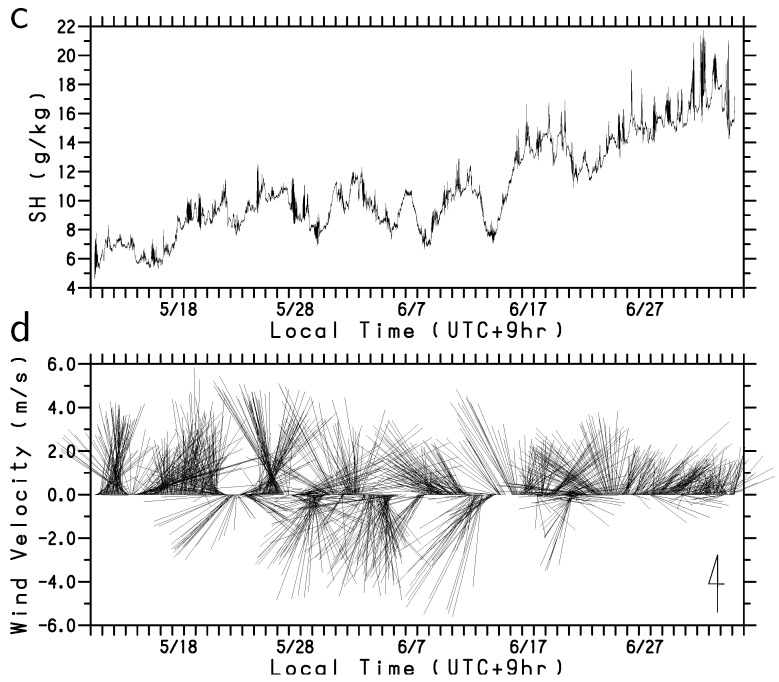
Time series of (**a**) SST (${}^{{}^{\circ}}$C), (**b**) air temperature (${}^{{}^{\circ}}$C), (**c**) specific humidity ($g\;{\mathrm{kg}}^{-1}$), and (**d**) wind velocity vector ($m\;s^{-1}$). The abscissa is local time (UTC+9h) during the observation period from 11 May to 5 July 2022.

The SST observed by the WG increased with time throughout the observation period (Figure 4a). Particularly, on 18 May 2022, when the WG crossed the SST front of the Oyashio south of Hokkaido, the SST increased significantly. An SST differential of more than approximately 5 ${}^{{}^{\circ}}$C can be observed across the front. Intriguingly, air temperature (Figure 4b) and specific humidity (Figure 4c) substantially increased along with the increase in SST, when the WG crossed the Oyashio front. Thus, the atmospheric boundary layer is significantly affected by the horizontal distribution of SST around the Oyashio.

Except for the period from mid-May to mid-June, the WG detected south to southwesterly winds (Figure 4d), consistent with the climatological wind direction in this season. The southerly winds along the northwestern rim of the North Pacific subtropical high transport moistened air mass from the south, e.g., [19], resulting in an increase in specific humidity (Figure 4c). During the period from mid-May to mid-June, atmospheric low-pressure systems encircled Japan and altered the wind speed and direction east of the Sanriku coast (Figure 4d). The passage of the low-pressure systems caused the atmospheric boundary layer to become cold and dry, as indicated by the low air temperature and low specific humidity in Figure 4b,c. The relationship between the low-pressure systems and turbulent heat flux will be discussed in detail below.

**Figure 5:**
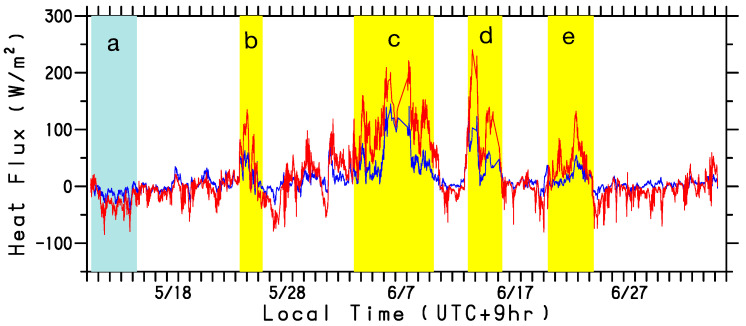
Time series of latent (red line) and sensible (blue line) sea surface heat fluxes ($W\;m^{-2}$) along the WG track. The abscissa is local time (UTC+9h) during the observation period from 11 May to 5 July 2022. Cyan and yellow bands marked by letters a–e indicate periods during which heat fluxes are shown in Figure 6.

In Figure 5, the LHF and SHF are represented by red and blue lines, respectively. Substantial heat transfer from the ocean to the atmosphere occurred during the yellow-banded periods of decreasing air temperature and specific humidity. Meanwhile, slight heat absorption by the ocean (cyan band in Figure 5) was observed during the period when the WG was stationed north of the Oyashio front (cyan band in Figure 4a) in the low SST region.

**Figure 6:**
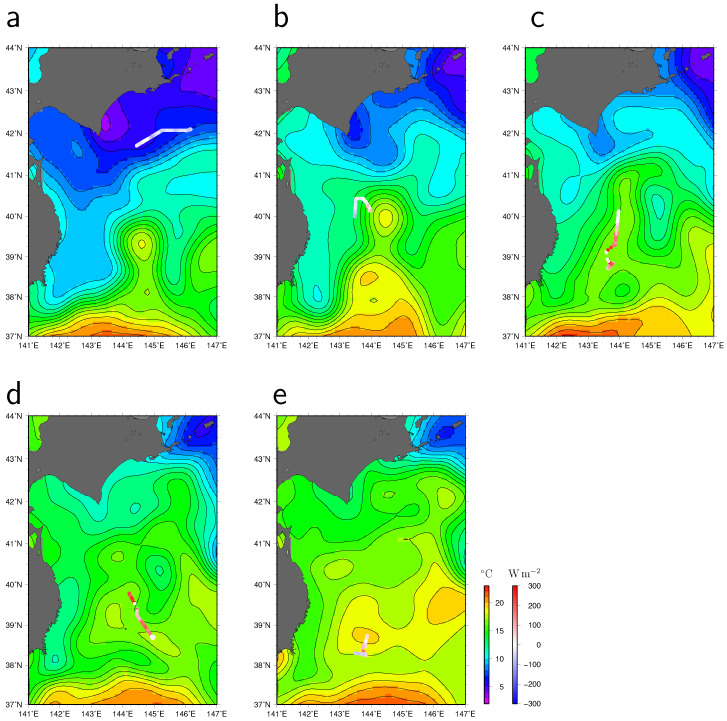
The Wave Glider tracks during the period shown by letters a–e in Figure 5, which correspond to panel letters of this figure. Values of turbulent (latent plus sensible) heat flux ($W\;m^{-2}$) are shown by colors of the WG tracks. Maps of SST (${}^{{}^{\circ}}$C) are satellite-observed values on (**a**) 13 May, (**b**) 25 May, (**c**) 7 June, (**d**) 15 June, and (**e**) 22 June 2022. The contour intervals of SST are 1 ${}^{{}^{\circ}}$C.

Maps of satellite-observed SST for individual heat absorption and release events marked, respectively, by cyan (a) and yellow (b–e) bands in Figure 5 are depicted in Figure 6. In early to mid-May, 2022, the coastal Oyashio water colder than 10 ${}^{{}^{\circ}}$C extended to just east of the Sanriku coast (Figure 6a). The warm Kuroshio water extended east of the Sanriku coast (Figure 6b,c) and reached the southern coast of Hokkaido (Figure 6d) after shedding an anticyclonic mesoscale eddy. i.e., a WCR, located approximately $39.4^{{}^{\circ}}$ N, $144.5^{{}^{\circ}}$ E from the KE (Figure 6a). In other words, as discovered by Hasegawa et al. [20], the cold coastal Oyashio current retreated to the east of Hokkaido, accompanied by the northward shedding of the WCR.

**Figure 7:**
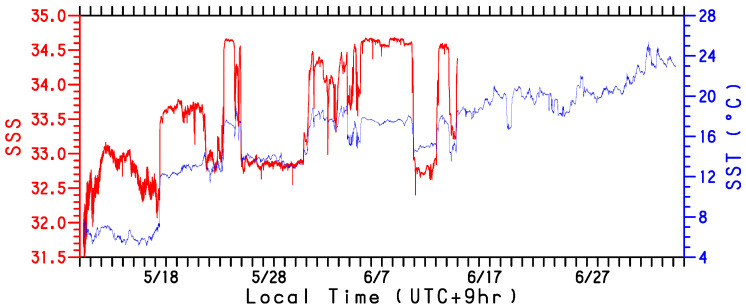
Time series of SSS (red line) in practical salinity scale along the WG track. The abscissa is local time (UTC+9h) during the observation period from 11 May to 5 July 2022. For comparison, SST (${}^{{}^{\circ}}$C) time series is shown by blue line.

The gradual increase in the observed SST by the WG after 1 June 2022 is partly attributable to the northward extension of the Kuroshio water. Although the WG had been in the warm Kuroshio water since 18 May, heat from the ocean was not always transferred to the atmosphere; instead, it was only deprived after when atmospheric disturbances passed over the WG (Figure 5). Furthermore, we identified high SST signals that are likely caused due to sub-mesoscale warm water regions in the Kuroshio water, such as those that occurred from 24 to 25 May, 31 May to 10 June, 13 to 14 June, and 21 to 23 June (Figure 4a). These signals correspond to high sea surface salinity (SSS) signals with the exception of 21 to 23 June (Figure 7). They cannot be fully detected by satellite observations because of the low resolution (Figure 6). The upward turbulent heat flux was notably large (>100 $W\;m^{-2}$ and >50 $W\;m^{-2}$ for LHF and SHF, respectively) in most of these sub-mesoscale high SST (>18 ${}^{{}^{\circ}}$C) regions (marked by letters b, c, d, and e in Figure 5). Although the WG was in very warm (~20${}^{{}^{\circ}}$C) water during 21 to 23 June (Figure 6e), the heat flux (letter e in Figure 5) was lower than in early and mid-June (letters c and d) because the air temperature and specific humidity were relatively high. This WG observation in the perturbed region revealed that sub-mesoscale warm waters provide significant heat to the atmosphere in late spring.

In accordance with Nagano et al. [6,10], we decompose the heat flux variation *F* into components due to SST ($T_{S}$), air temperature ($T_{A}$), specific humidity (*q*), and wind speed (*U*), in order to examine the variables that are responsible for the variations in LHF and SHF, as follows:

(1) $$\begin{array}{c} \Delta F\;\approx \;\left(\frac{\partial F}{\partial T_{S}}\right)\Delta T_{S}+\left(\frac{\partial F}{\partial T_{A}}\right)\Delta T_{A}+\left(\frac{\partial F}{\partial q}\right)\Delta q+\left(\frac{\partial F}{\partial U}\right)\Delta U, \end{array}$$

where $\Delta T_{S}$, $\Delta T_{A}$, Δq, and ΔU are temporal variations in SST, air temperature, specific humidity, and wind speed, respectively. Due to the complexity of the turbulent heat flux function, the rate of change of each term on the right-hand side of Equation (1) was determined using the mean values of the other three variables during the observation period. Units of all the terms of Equation (1) are the same as that of turbulent heat flux, i.e., $W\;m^{-2}$.

**Figure 8:**
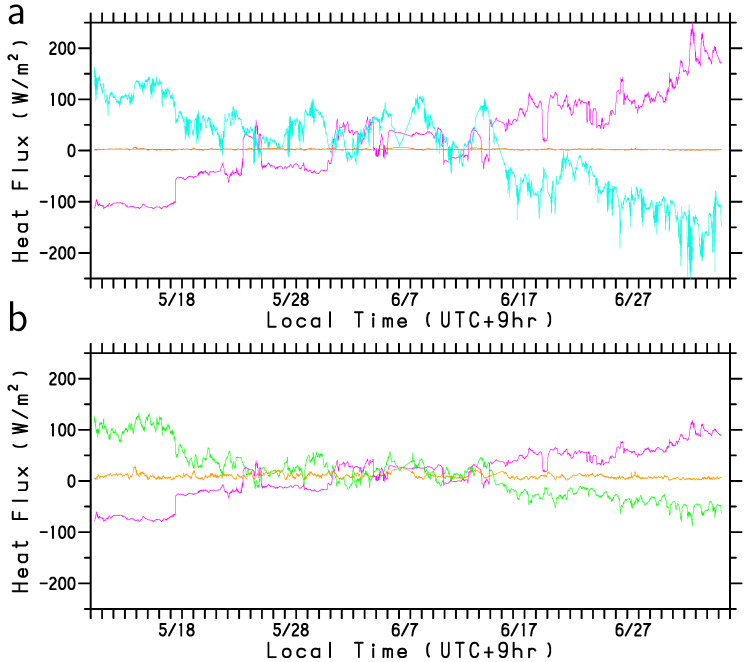
(**a**) LHF and (**b**) SHF variation components ($W\;m^{-2}$) in Equation (1). The components of SST, air temperature, specific humidity, and wind speed are shown by magenta, green, cyan, and orange lines, respectively. Because SHF is independent of specific humidity, the SHF variation due to specific humidity is not plotted in panel (**b**). The abscissa is local time (UTC+9hr) during the observation period from 11 May to 5 July 2022.

The variation components in LHF and SHF due to the four variables are depicted in Figure 8a,b, respectively. However, the wind speed variation was not primarily responsible for the increase in turbulent heat flux (orange lines in Figure 8). This is because the strong winds were accompanied by a relatively high air temperature (Figure 4b) and high specific humidity (Figure 4c). Throughout the observation period, the effects of the increase in SST (magenta line in Figure 8) on LHF and SHF were canceled out by the increases in specific humidity (Figure 8a) and air temperature (Figure 8b). The enhancements of the LHF (red line in Figure 5) and SHF (blue line) due to the atmospheric disturbances such as from 24 to 25 May, 1 to 10 June, and 13 to 15 June are attributed to the positive effects of specific humidity (~50–100 $W\;m^{-2}$) (cyan line in Figure 8a) and air temperature (~10–50 $W\;m^{-2}$) (green line in Figure 8b) in addition to the positive effect of sub-mesoscale high SST regions (magenta lines in Figure 8). The effect of sub-mesoscale high SST waters was variable up to ~50 $W\;m^{-2}$ (~30 $W\;m^{-2}$) for the LHF (SHF) variation. Thus, although this humidity effect may be underestimated as described in Section 2, the humidity decreases are found to be particularly important for the enhancement of LHF in late spring.

**Figure 9:**
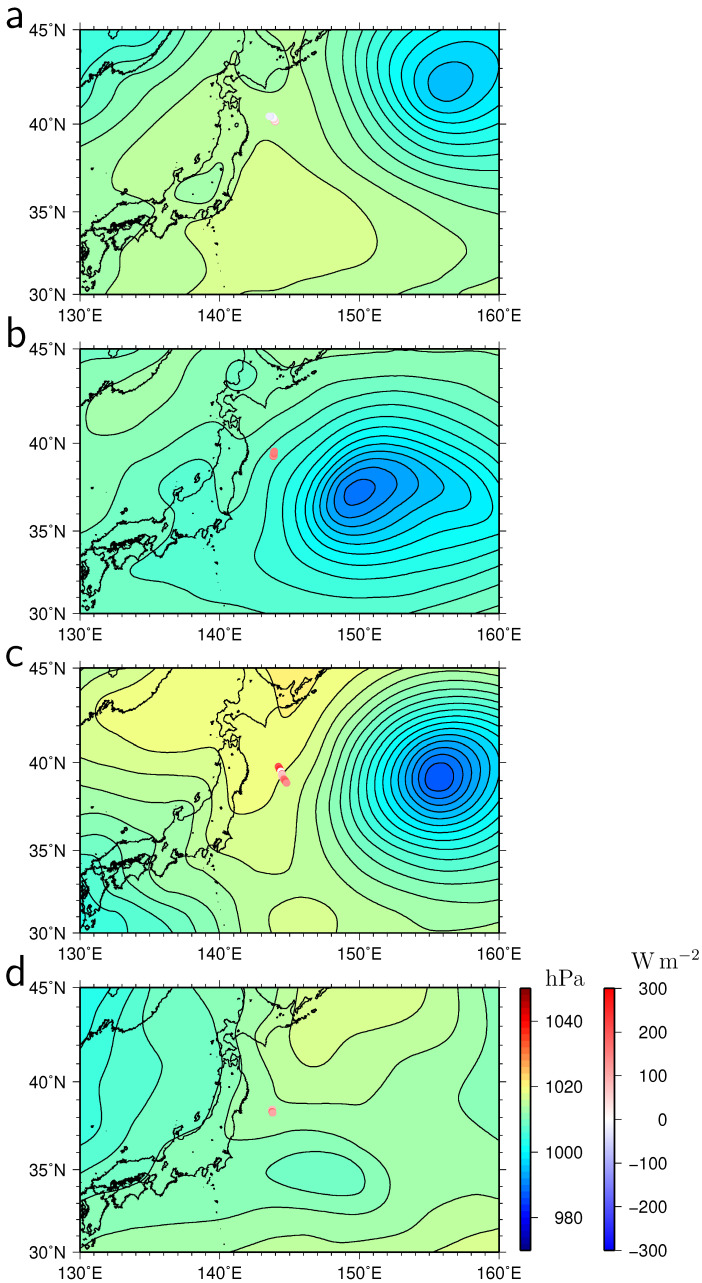
Maps of sea-level pressure (hPa) on (**a**) 24 May, 15:00, (**b**) 7 June, 21:00, (**c**) 13 June, 21:00, and (**d**) 23 June, 03:00, 2022 around the positive peaks of the turbulent (latent plus sensible) heat flux. The contour intervals of sea-level pressure are 2 hPa. The WG tracks around the heat flux peak periods are shown by lines colored by values of heat flux ($W\;m^{-2}$).

Figure 9 illustrates sea-level pressure maps during the peak periods of the turbulent heat flux. The turbulent heat flux was positively large (marked by letters by b, c, and d in Figure 5) except on 23 June 2022, when the atmospheric low-pressure systems proceeded to the east of the WG and wind blew southward (Figure 9a–c). Although the winds were strong during the passage of these low-pressure systems, high air temperature and specific humidity prevented heat from being released into the atmosphere. Prior to the rise of the turbulent heat flux (letter c in Figure 5), the SSS was low and highly variable from 1 to 4 June (Figure 7), occasionally reaching below 33. The low-SSS signal corresponds to the passage of the low pressure and may be the result of heavy rainfall. This is consistent with the high specific humidity. Due to the high atmospheric pressure to the northeast of Hokkaido on June 23 (Figure 9d), relatively dry and low-temperature winds blew from the east and southeast as observed by the WG (Figure 4b,c), releasing heat into the atmosphere (letter e in Figure 5).

## 4. Summary and Conclusions

In the region off the Sanriku coast of Japan, also known as the perturbed waters from the Kuroshio and Oyashio, are distributed in a convoluted manner. In addition to the mesoscale WCRs shed by the KE, smaller scale (sub-mesoscale) warm water regions exist in the perturbed area; however, these such sub-mesoscale features cannot be detected clearly by satellites. The very cold dry wind from the Eurasian Continent is known to deprive a huge amount of heat from the Kuroshio water, which contributes to the global heat balance. Heat fluxes over such sub-mesoscale warm waters in other seasons may affect Japan’s regional climate and the weather. In this study, we focused on the atmosphere and ocean in late spring, the humidity variable season. Due to the potential for research vessels to affect the ocean and atmosphere boundary layers, smaller platforms are preferred for estimating turbulent heat flux. Therefore, we observed the atmosphere and ocean using a wave-propelled autonomous surface vehicle called a Wave Glider that was developed by Liquid Robotics.

In addition to meteorological sensors installed by default for measuring the wind speed/direction and air temperature, we equipped the WG with CTD JES10mini and HOBO air temperature/relative humidity sensors. By covering the HOBO sensors with Gore-Tex fabric, we prevented the sensors from being damaged by seawater immersion. The WG was deployed on 11 May 2022 and was recovered on 5 July 2022. As a result, we were able to measure atmospheric and oceanic data for 55 days despite the severe weather conditions of several atmospheric low-pressure system crossings. The variation amplitude in the air temperature detected by the HOBO sensor covered by the Gore-Tex fabric was found to be 77% of that observed by the Weather Station sensor. Therefore, the variation in the specific humidity measured by the HOBO sensor may be underestimated by approximately 33%.

Throughout the observation period, the air temperature increased with time along with the seasonal increase in SST. Along the northwestern rim of the North Pacific subtropical high, a southerly wind transported moist air, resulting in a rise in the specific humidity prior to and in the Baiu season. Beyond seasonal variations, the WG also detected signals of different origin. Particularly, the WG reported an SST increase of more than 5 ${}^{{}^{\circ}}$C when passing over the Oyashio SST front. Because of the increase in SST, the air temperature and specific humidity also increased. After entering the warm Kuroshio water zone, the WG detected warmer and saltier sub-mesoscale water regions. Cold dry air masses advected by northerly winds after the passage of atmospheric low-pressure systems were observed as the air temperature and specific humidity decreased.

We estimated LHF and SHF from the atmospheric and oceanic variables obtained from the WG observation using the bulk flux algorithm. The heat flux variations were mostly caused by variations in air temperature and specific humidity rather than variations in wind speed. Although the humidity variation is possibly underestimated, we obtained the turbulent heat flux variation due to the humidity variation over ocean mesoscale and sub-mesoscale warm waters in the perturbed area. The LHF and SHF were slightly downward in the low SST region north of the Oyashio front. Substantially upward LHF and SHF were induced as cold dry air masses were advected to over sub-mesoscale warm water regions, primarily by northerly winds following the passage of atmospheric disturbances, whereas no significant heat flux was produced in the surrounding Kuroshio water region.

These results imply that heat flux studies only based on the satellite data possibly underestimate the impacts of ocean sub-mesoscale variations on the atmosphere because turbulent heat fluxes due to sub-mesoscale variations cannot be monitored by satellite data because of the low spatial resolution. In addition to wide-coverage observations by satellites, high-resolution observations by USVs are required in the future to obtain more exact knowledge on the role of the oceanic mesoscale and sub-mesoscale variations in the regional climate and the weather and are also useful to improve climate and weather forecasts.

## Figures and Tables

**Table 1:** Information of meteorological and oceanographic instruments installed to the Wave Glider.

Measurement	Model	Manufacturer	Accuracy	Resolution
Wind Speed	200WX	AIRMAR	±5%	0.1 m $s^{-1}$
Wind Direction	Weather	Technology	±3 ${}^{{}^{\circ}}$	0.1 ${}^{{}^{\circ}}$
Air Pressure	Station		±0.5 hPa	0.1 hPa
Air Temperature			±1.1 ${}^{{}^{\circ}}C$	0.1 ${}^{{}^{\circ}}C$
				
Air Temperature	HOBO U23	Onset	±0.2 ${}^{{}^{\circ}}C$	0.04 ${}^{{}^{\circ}}C$
Relative Humidity	Pro v2	Computer	±2.5%	0.05%
	Data Logger			
				
				
Water Temperature	JES10mini	Offshore	±0.005 ${}^{{}^{\circ}}C$	0.0001 ${}^{{}^{\circ}}C$
Conductivity		Technologies	±0.005 $S\;m^{-1}$	0.00001 $S\;m^{-1}$
Water Pressure			±0.1% FSR	

## Data Availability

NOAA OI SST V2 High-Resolution Dataset was obtained from the website of NOAA Physical Science Laboratory (https://psl.noaa.gov/data/gridded/index.html, accessed on 9 December 2022). JRA-55 sea-level pressure data were provided by Center for Computational Sciences, University of Tsukuba (http://gpvjma.ccs.hpcc.jp/~jra55/index.html, accessed on 9 December 2022).

## Abbreviations

The following abbreviations are used in this manuscript:

CTD	Conductivity-temperature-depth
AP	Air pressure
AT	Air temperature
FSR	Full-scale range
GNSS	Global navigation satellite system
JRA-55	Japanese 55-year Reanalysis
JST	Japanese standard time
KE	Kuroshio Extension
LHF	Latent heat flux
NOAA	National Oceanic and Atmospheric Administration
OI	Optimal interpolation
RH	Relative humidity
R/V	Research vessel
SH	Specific humidity
SHF	Sensible heat flux
SSS	Sea surface salinity
SST	Sea surface temperature
USV	Unmanned surface vehicle
WCR	Warm-core ring
WG	Wave Glider
